# Anti-tumor effect of Liqi, a traditional Chinese medicine prescription, in tumor bearing mice

**DOI:** 10.1186/1472-6882-9-20

**Published:** 2009-07-01

**Authors:** Deng-Bo Ji, Jia Ye, Yi-Min Jiang, Bo-Wen Qian

**Affiliations:** 1 Department of Molecular and Cellular Pharmacology, School of Pharmaceutical Sciences, Peking University, Beijing, 100191, PR China; 2 Medical and Healthy Analysis Center, Peking University, Beijing, 100191, PR China; 3 Department of Traditional Chinese Medicine, Shanghai University of Chinese Traditional Medicine, Shanghai, 201203, PR China

## Abstract

**Background:**

*Liqi*, an herbal preparation used in traditional Chinese medicine, has been used to treat cancer in China for centuries. We investigated the anti-tumor effects of liqi and their mechanisms in mice that had been xenografted with tumors.

**Methods:**

Sarcoma 180 tumor, Lewis lung carcinoma, and SGC-7901 cells were implanted in BALB/c mice, C57BL/6 mice, and BALB/c nude mice, respectively. Liqi was administered to subgroups of these mice. The tumor weight and size were measured. Cell cycle analysis and T lymphocyte subsets were determined by flow cytometry. The activity of NK cells and TNF was tested using cytotoxicity assay on YAC-1 cells and L929 cells, respectively, and the activity of IL-2 was tested with an IL-2-dependent CTLL-2 cell proliferation assay. Platelet aggregation was monitored by measuring electric impedance, and the levels of thromboxane A2 (TXA_2_) and prostacyclin (PGI_2_) in blood were measured by ^125^I-TXB_2 _and ^125^I-Keto-PGF_1α _radioimmunoassay.

**Results:**

The results showed that liqi inhibited tumor growth in tumor-implanted mice and arrested the cell proliferation in the G0/G1 phase and reduced the portion of cells in S and G2/M phase for SGC-7901 cells. Liqi increased the activity of NK cells and TNF-α, stimulated IL-2 production and activity, and regulated T lymphocyte subpopulations. Liqi inhibited the Lewis lung carcinoma metastasis by inhibiting platelet aggregation and normalizing the balance between TXA_2 _and PGI_2_.

**Conclusion:**

All these findings demonstrated that liqi has an anti-tumor effect in vivo. The mechanism may be related to immune regulation and anticoagulation effects.

## Background

Cancer is a leading cause of death worldwide. The main curative therapies for cancer are surgery and radiation, although these are most successful if the tumor is diagnosed at an early stage. For advanced tumors, chemotherapy is the treatment of choice, and although these drugs are effective, they are associated with severe adverse events and drug resistance [1], and new therapeutic options are needed. In the search for new cancer therapeutics with low toxicity and few side effects, traditional Chinese medicines are promising candidates.

*Liqi *prescription, a traditional Chinese medicine made from *Poncirus trifoliate (L.)Raf, Akebia Trifoliate Koidz, Citrus medica var. sarcodactylis Swingle *and *Saussurea lappa*, has been used to treat malignancies in traditional Chinese medicine for centuries. The main active constituents are coumarins, flavonoids, and terpenoids in *Poncirus trifoliate (L.)Raf*; triterpene and triterpenoid saponin in *Akebia Trifoliate Koidz; *terpene, flavonoids and polysaccharide in *Citrus medica var. sarcodactylis Swingle; *and sesquiterpenes and sesquiterpene lactones in *Saussurea lappa*[2-5]. Recent studies have demonstrated their multiple pharmacologic actions, including anti-inflammatory, anti-allergic and antibacterial effects; promoting gastrointestinal motor function; and inhibiting cancer cell growth in vitro. *Poncirus trifoliate *inhibits human colon cancer HT-29 cell growth and induces human leukemia HL-60 cell apoptosis [6,7]. *Akebia Trifoliate Koidz *inhibits the growth of breast cancer cell lines MDA-MB-231 and BT-483[8]. *Saussurea lappa *induces G2-growth arrest and apoptosis in AGS gastric cancer cells [9].

However, little research has been conducted on the anti-tumor effects of liqi in vivo. In the present experiment, we studied the anti-tumor effect of liqi and explored the mechanism of any effect on tumor-bearing mice.

## Methods

### Materials

*Poncirus trifoliate (L.)Raf, Akebia Trifoliate Koidz, Citrus medica var. sarcodactylis Swingle *and *Saussurea lappa *were purchased from Shanghai Pharmaceutical Co., Shanghai, China, and were authenticated by Prof. Bo-Wen Qian, Department of Traditional Chinese Medicine, Shanghai University of Chinese Traditional Medicine, Shanghai, China. We also used the following materials: Concanavalin A (ConA), lipopolysaccharide (LPS), propidium iodide, ADP (Sigma, St Louis, MO); [^3^H]TdR (China Institute of Atomic Energy, Beijing, China); human recombinant interleukin-2 (rhIL-2) (Boehringer Mannhein GmbH, Germany); human recombinant tumor necrosis factor (TNF) (Collaborative Biomedical Products, Bedford, MA); FITC-conjugated antibodies to CD3, CD4, CD8 (Pharmingen, Becton Dickinson, Franklin Lakes, NJ); lactic dehydrogenase (LDH) Cytotox assay kit (Promega, Madison, WI); ^125^I-TXB_2 _and ^125^I-Keto-PGF_1α _radioimmunoassays (RIA kits, Suzhou Medical College, Suzhou, China); RPMI Medium 1640 (Invitrogen, Carlsabad, CA); fetal bovine serum (FBS, HyClone Corporation, Logan, UT).

### Cell culture

Mouse sarcoma 180 tumor cells, Lewis lung carcinoma cells, and human gastric carcinoma cell line SGC-7901 were donated by Shanghai Institute of Materia Medica, Chinese Academy of Sciences (Shanghai, China). YAC-1, CTLL-2, and L929 cells were provided by School of Medicine, Shanghai Jiao Tong University (Shanghai, China), and were grown routinely in RPMI-1640 supplemented with 10% heat-inactivated fetal bovine serum (FBS). The medium was supplemented with 100 U/mL penicillin and 100 U/mL streptomycin; 10 ng/mL rhIL-2 was added in the culture medium of CTLL-2 cells, and the cells were incubated in a humidified atmosphere, with 5% CO_2 _in air at 37°C.

### Animals

Male BALB/c mice and C57BL/6 mice (6 to 8 weeks old) were provided by the Department of Shanghai Institute of Planned Parenthood Research, Fudan University, Shanghai, China. The animals were group-housed in a regulated environment (22 ± 1°C, relative humidity 60 ± 5%) with a 12-h light and 12-h dark cycle (08:00–20:00, light). Food and water were given *ad libitum*. Food pellets meet Feed Standard of Medical Laboratory Animal of China. Food composition is as follows: protein18–25%, fat 4–5%, calcium 1.0–1.8%, phosphonium0.6–1.2%, vitamins A 12500–15000 IU/kg, vitamins D 1250–1500 IU/kg, fiber 4–5% and moisture 8–10%, lysine 0.98–1.42%, cystine0.76–1.10%, tryptophane 0.22–0.34%.

BALB/c strain athymic nu/nu mice were provided by Shanghai Cancer Institute, China. Female mice (6 to 8 weeks old) were used in this study. The animals were maintained in a specific pathogen free animal care facility, under controlled conditions (25 ± 2°C, 50%–60% relative humidity and 12-hour light cycle). They were fed with autoclaved tap water and food *ad libitum*. The laboratory animal protocol used for this study was approved by the committee for control and supervision of experimental animals of Shanghai University of Chinese Traditional Medicine.

### Preparation of Liqi

Liqi was prepared as a lyophilized powder of hot water extracts from 4 species of medical herbs: *Citrus medica var. sarcodactylis Swingle, Akebia Trifoliate Koidz, Poncirus trifoliata(L.)Raf *and *Saussurea lappa*. Briefly, the above materials were mixed in a ratio of 3:2:1.7:1.7 and decocted three times with boiling distilled water for 1 h. The decoction was filtered, collected, concentrated, and lyophilized. The yield of liqi was approximately 20%. Liqi was dissolved in distilled water and administered in a volume of 10 ml/kg. In this experiment, the animal dose of liqi was dose of crude drug.

### Tumor models and in vivo treatment regimen

Sarcoma 180 tumor cells were subcutaneously implanted (2 × 10^6 ^S180 tumor cells suspended in 50 μl of Ca^2+^- and Mg^2+^-free phosphate-buffered saline [PBS]) in the right axillary region of syngeneic BALB/c mice. Lewis lung carcinoma cells were subcutaneously implanted (2 × 10^6 ^LLC tumor cells suspended in 50 μl of PBS) in the right axillary region of syngeneic C57BL/6 mice. SGC-7901 cells were subcutaneously implanted (2×10^5 ^cells suspended in 0.2 ml of PBS) in the right axillary region of BALB/c nude mice. After 24 h, they were weighed and randomized into 5 groups (12 mice each): a normal control group, a group that was injected with tumor cells but treated with water only, and three groups that were injected with tumor cells and treated with different doses of liqi (12.5, 25 and 50 g/kg). Treatments were administrated at about 9 o'clock daily by gavage, and mice were weighted daily.

### Anti-tumor activity

On day 21, all mice were weighed and euthanized, and tumors were removed and weighed. The tumor repression rate was calculated as follows: The repression rate (%) = (mean weight of tumors of water-treated mice – mean weight of tumors of liqi-treated mice)/mean weight of tumors of water-treated mice × 100% [10].

### Cell cycle analysis of SGC-7901 cells from xenografted mice

For cell cycle analysis, cells were harvested from xenografted BALB/c nude mice. On day 21, tumor tissues were immediately removed and disaggregated in PBS and filtered through a double layer of stainless-steel mesh using a syringe plunger to obtain single cell suspension. Cell cycle distribution was evaluated by propidium iodide staining of nuclei and flow cytometric analysis [11]. Briefly, pelleted cells were washed twice with PBS and then fixed in 70% cold ethanol overnight at -20°C. After washing again, the cells were resuspended in PBS containing RNase A (200 μg/mL) and incubated at 37°C for 30 min. Propidium iodide was added to the cell suspensions at a final concentration of 100 μg/mL. The fluorescence intensity of PI was analyzed with a FACS calibur flow cytometer (FACS Calibur; Becton Dickinson, USA) and Cell Quest software.

### Flow cytometry for peripheral blood T lymphocyte subsets

On day 12, the heparinized peripheral blood was collected from the orbital plexuses of the tumor-bearing C57BL/6 mice to analyze peripheral blood lymphocyte subsets by flow cytometry. Samples were prepared by adding 5 μl of fluorescent monoclonal antibodies against CD3, CD4, and CD8 to 100 μl of heparinized whole blood. CD3+CD4+ (T helper cells) and CD3+CD8+ (T suppressor cells) were counted. Lymphocytes were separated from erythrocytes lysed. The tubes were placed on ice in the dark until flow cytometric analysis; five thousand cells were collected for each sample, and data were analyzed by using flow cytometry.

### IL-2 activity assay

In order to determine the effects of Liqi on IL-2 production and activity, splenic lymphocytes from tumor bearing BALB/c mice were prepared after treatment with liqi (50 g/kg) for 12 days as described above. Spleens of mice were removed under sterile conditions and disaggregated in PBS and filtered through a double layer of stainless-steel mesh using a syringe plunger to obtain single-cell suspension. Lymphocytes were collected and suspended in RPMI-1640 medium at a concentration of 1×10^7 ^cells/mL, with ConA added to a final concentration of 5 μg/mL. After incubating at 37°C and 5% CO_2 _for 24 hours, cells supernatants containing extracellular IL-2 were collected and stored at -20°C until assay.

IL-2 activity was tested using an IL-2-dependent CTLL-2 cell proliferation assay [12]. The CTLL-2 cells were washed in medium containing 2% FBS and incubated for 2 h without IL-2. The cells were then cultured (1× 10^5 ^cells/mL) in 96-well plates with 0.6 ng/mL of rhIL-2 or with spleen cell supernatant for 24 h. Six hours before the end, 0.5 μCi [^3^H]TdR 50 μL was added to each well. The [^3^H]TdR incorporation was measured with a liquid-scintillator (Beckman Co, USA) counting technique. The results were described as the average of triplicate Bq (specific radioactivity of [^3^H]TdR was 20 μCi/mmol).

### Evaluation of NK cell cytotoxicity

NK cells from tumor bearing BALB/c mice spleens were prepared as described above and used as effector cells. YAC-1 cells, mouse lymphoma sensitive to NK cells were used as target cells. Effector and target cells resuspended in RPMI-1640 medium were added to each well of a 96-well round-bottom microculture plate in triplicate to obtain an effector/target (E/T) ratio of 100:1 and incubated for 16 h. The amount of released lactate dehydrogenase (LDH) in culture supernatants was determined using the LDH Cytotox assay kit according to the manufacturer's instructions. The OD was read at 490 nm with a Microplate Reader. The percentage of NK cell cytotoxicity was calculated with the formula: cytotoxicity (%) = (experimental release – effector spontaneous release – target spontaneous release)/(target maximum release – target spontaneous release) × 100. Experimental release stood for LDH that was released from cocultures at an E/T ratio of 100:1, effector spontaneous release or target spontaneous release was spontaneous LDH release from effector or target cells incubated with medium alone, and target maximum release was obtained from target cells lysed with the lysis solution [13].

### Production and activity assay of TNF

On day 12, tumor-bearing BALB/c mice received 20 μg/0.2 ml of LPS intravenously. After 1.5 h, blood samples were collected from the orbital plexuses of the mice. The sera were stored at -20°C until used for the TNF assay. TNF activity was determined by a cytotoxicity assay on L929 cells [14]. Briefly, L929 cells were plated at the density of 3.5 × 10^4^/ml in 96-well plates and incubated at 37°C in a 5% CO_2 _atmosphere for 24 h. Samples or recombinant TNF (as the standard) were incubated for a further 24 h, in the presence of 2 μg/ml actinomycin D. Cell survival was determined by measuring optical density at 630 nm after crystal violet staining. The diluted samples were assayed in triplicate. The percentage of cell destruction at a particular dilution was calculated as (A_cont _– A_dil_/A_cont_) × 100, where A_cont _is absorbance in control wells and A_dil _is absorbance in wells of a particular dilution of sample. The percent cytotoxicities were plotted against the logarithm of sample quantity. One unit of TNF activity was defined as the sample quantity required to achieve 50% cytotoxicity in the reaction.

### Inhibition of Metastasis in Mice

The tumor-bearing C57BL/6 mice were sacrificed on the 21st day. Lungs were removed and fixed in 4% formalin. Tumor colonies were counted under microscope (× 200).

### Platelet Aggregation Assay

Platelet aggregation was monitored by measuring electric impedance [15] using a whole-blood aggregometer (model OX-200; Shanghai Medical University Instrument Factory, Shanghai, China). On day 21, heparinized blood was drawn from tumor-bearing C57BL mice by cardiac puncture. Whole blood was then diluted with an equal amount of normal saline. The sample was placed in a plastic cuvette containing a magnetic stir bar and was kept at 37°C for 5 minutes before analysis. The platelet aggregation was then initiated by adding 10 μl 2 μmol/L ADP and monitored for up to 5 minutes.

### Measurement of TXA_2 _and PGI_2_

On day 21, blood samples were collected from the femoral artery of the tumor-bearing C57BL/6 mice and transferred into a plastic tube prefilled with EDTA-Na_2 _(9:1). The tube was centrifuged at 1200 × g at 4°C for 10 min. The supernatant was frozen at -20°C. TXA_2 _with a half-life of approximately 30 sec under physiologic conditions was measured using its stable metabolite TXB_2_, and PGI_2 _using its stable metabolite 6-keto-prostaglandin F1α [16]. All determinations were performed using commercially available ^125^I-TXB_2 _and ^125^I-Keto-PGF1α radioimmunoassays (RIA kits, Suzhou Medical College, Suzhou, China).

### Statistical analysis

The data are expressed as mean ± S.D. Student's t-test or Dunnet *t*-test was used to compare the differences between treated groups and control groups, and differences were considered significant at P < 0.05.

## Results

### Effect of liqi on tumor growth in the tumor-transplanted mice

To explore the role of liqi on tumor growth in mouse Sarcoma 180 transplanted into BALB/c mice and Lewis lung carcinoma transplanted into C57BL/6 mice, we treated tumor-bearing mice with various doses of liqi (12.5, 25, 50 g/kg). For Sarcoma 180-bearing BALB/c mice, liqi significantly decreased the tumor growth, with 29.69% inhibition at 25 g/kg and 34.54% inhibition at 50 g/kg (*p < 0.05*, Table 1). For Lewis lung carcinoma-bearing C57BL/6 mice, liqi significantly decreased the tumor growth, with 35.20% inhibition at 25 g/kg and 42.54% inhibition at 50 g/kg (*p < 0.05*, Table 2). There was no significant difference between the body weight of the liqi-treated group and that of the normal group.

**Table 1:** Effect of Liqi on mouse Sarcoma 180 transplanted into BALB/c mice

**Groups**	**Doses (g/kg)**	**n**	**Mean weight of tumor (g)**	**Inhibition (%)**
Normal	0	12	0	0
Model	0	12	1.65 ± 0.47	0
Liqi	12.5	12	1.28 ± 0.50	22.42%
	25	12	1.16 ± 0.49*	29.69%
	50	12	1.08 ± 0.50*	34.54%

Each group consists of 12 mice. Results are presented as mean ± S.D. *P < 0.05, compared with model group.

**Table 2:** Effect of Liqi on mouse Lewis lung carcinoma transplanted into C57BL/6 mice.

**Groups**	**Doses (g/kg)**	**n**	**Mean weight of tumor (g)**	**Inhibition (%)**
Normal	0	12	0	0
Model	0	12	1.57 ± 0.62	0
Liqi	12.5	12	1.11 ± 0.51	29.06%
	25	12	1.02 ± 0.48*	35.20%
	50	12	0.90 ± 0.44*	42.54%

Each group consists of 12 mice. Results are presented as mean ± S.D. *P < 0.05, compared with model group.

**Figure 1:**
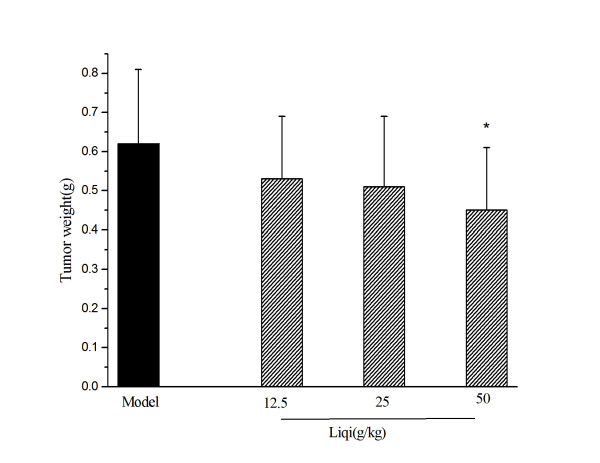
**Effect of Liqi on SGC7901 transplanted into BALB/c nu/nu mice**. SGC-7901 cells were subcutaneously implanted (2 × 10^5 ^cells suspended in 0.2 ml of PBS) in the right axillary region of BALB/c nude mice. After 24 h, Liqi (12.5, 25, 50 g/kg) was administrated daily by gavage for 21 days. The tumor weight was measured. Results are expressed as the mean ± S.D. of 12 mice. *P < 0.05, compared with untreated control of tumor-transplanted animals (model group).

To determine whether liqi could suppress human gastric carcinoma growth in vivo, we implanted SGC-7901 in nude mice. As shown in Figure 1, Liqi (50 g/kg) reduced the tumor weight significantly compared with the model group (*p < 0.05*), with inhibition 27.42%.

### Effect of Liqi on SGC-7901 cells cycle from xenografted mice

As shown in Table 3, cell cycle analysis showed accumulation of SGC-7901 cells in the G0/G1 phase (*p < 0.01*) and decreased cell numbers in the S (*p<0.01*) and G2/M phases in mice treated with 50 g/kg of liqi.

**Table 3:** Effect of Liqi on cell cycle of SGC-7901 cells from xenografted mice.

**Groups**	**Dose(g/kg)**	**n**	**Cell cycle (%)**		
			
			**G1**	**S**	**G2+M**
Model	0	7	49.7 ± 6.8	44.9 ± 6.6	5.4 ± 0.76
Liqi	50	7	61.8 ± 6.4**	34.0 ± 5.3**	4.13 ± 1.2

Each group consists of 7 mice. Results are presented as mean ± S.D. **P < 0.01, compared with model group.

### Effect of liqi on T lymphocyte subpopulations in tumor-bearing mice

**Figure 2:**
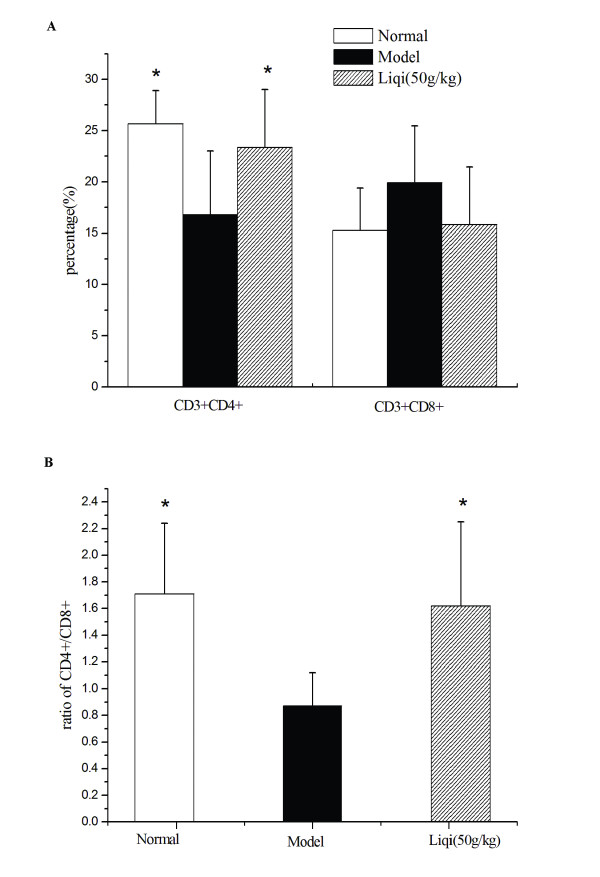
**Effect of Liqi on T lymphocyte subpopulations in tumor-bearing mice**. Sarcoma 180 tumor cells were subcutaneously implanted (2 × 10^6 ^cells suspended in 50 μl of PBS) in the right axillary region of syngeneic BALB/c mice. After 24 h, Liqi (50 g/kg) was administrated daily by gavage for 12 days. Peripheral blood lymphocyte subsets were measured by flow cytometry. Results are expressed as the mean ± S.D. of 12 mice. *P < 0.05, compared with untreated control of tumor-transplanted animals (model group). The normal group indicates healthy animals without tumor xenograft for comparison.

As shown in Figure 2, the numbers of CD3+ CD4+ T cells and the CD4+/CD8+ ratios in peripheral blood were consistently lower in the tumor-bearing mice than in the normal mice (*P *< 0.05). Among tumor-bearing mice, those treated with liqi had consistently higher percentages of CD3+CD4+ T lymphocytes than did untreated mice (*P *< 0.05). A similar difference was seen in the ratios of CD4+/CD8+; however, the percentages of CD3+CD8+ T lymphocytes were slightly lower in liqi-treated mice.

### Effect of liqi on IL-2 activity in tumor-bearing mice

**Figure 3:**
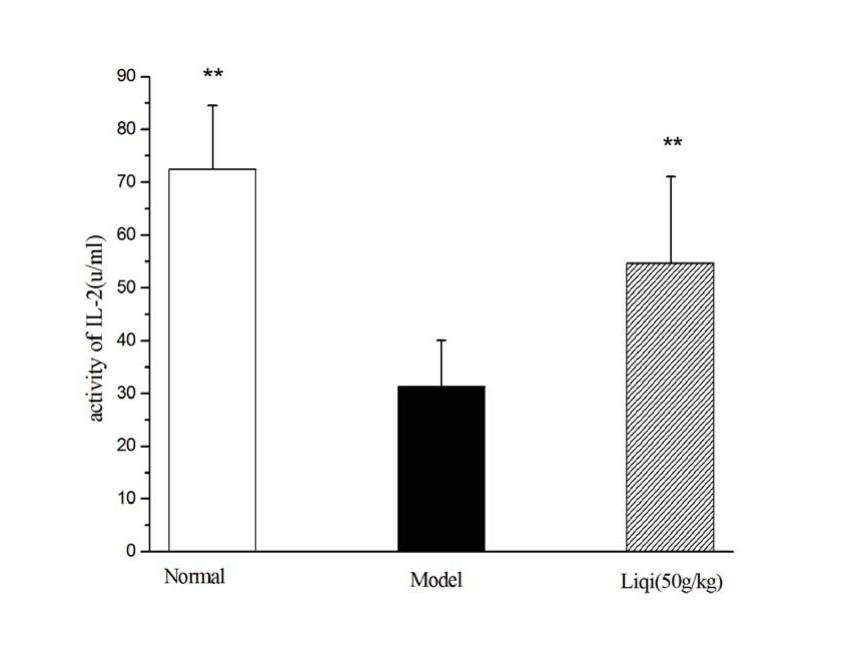
**Effect of Liqi on IL-2 activity in tumor-bearing mice**. Sarcoma 180 tumor cells were subcutaneously implanted (2 × 10^6 ^cells suspended in 50 μl of PBS) in the right axillary region of syngeneic BALB/c mice. After 24 h, liqi (50 g/kg) was administrated daily by gavage for 12 days. IL-2 production and activity in splenocyte supernatants were tested using an IL-2-dependent CTLL-2 cell proliferation assay. Results are expressed as the mean ± S.D. of 12 mice. **P < 0.01, compared with untreated control of tumor-transplanted animals (model group). The normal group indicates healthy animals without tumor xenograft for comparison.

To illustrate whether liqi could affect the IL-2 production and activity in splenocytes, the murine T-cell line CTLL-2 was used. As shown in Figure 3, splenocytes supernatants from tumor-bearing mice inhibited the proliferation of CCLL-2 cells compared with the normal group (P < 0.01). After treatment with liqi (50 g/kg), splenocyte supernatants showed a greater proliferation of CCLL-2 cells than in splenocytes from untreated mice (P < 0.01).

### Effect of liqi on NK cell cytotoxicity in tumor-bearing mice

**Figure 4:**
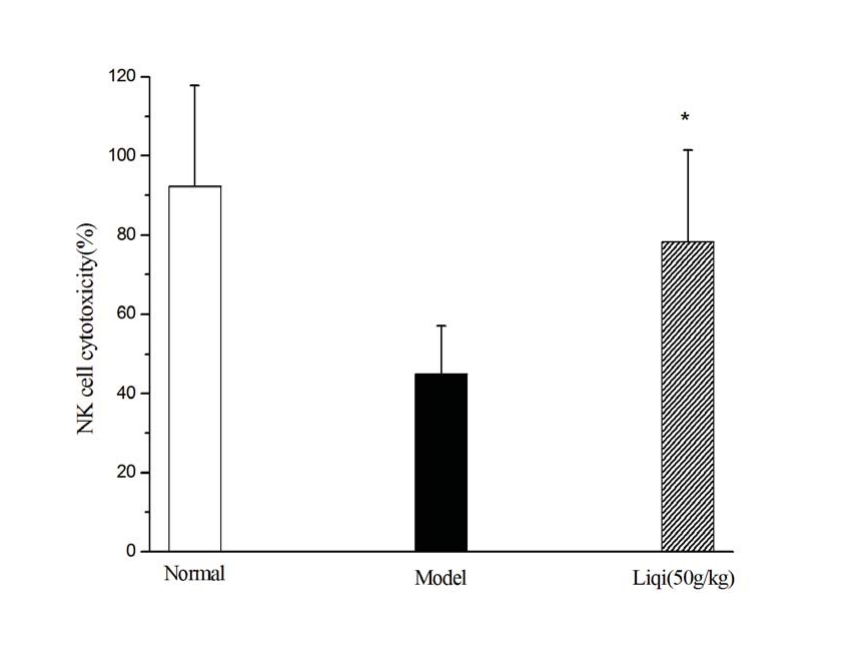
**Effect of Liqi on NK cell cytotoxicity in tumor-bearing mice**. Sarcoma 180 tumor cells were subcutaneously implanted (2 × 10^6^cells suspended in 50 μl of PBS) in the right axillary region of syngeneic BALB/c mice. After 24 h, liqi (50 g/kg) was administrated daily by gavage for 12 days. NK cell cytotoxicity was tested using YAC-1 cells as target cells. LDH in culture supernatants was determined using the LDH Cytotox assay kit according to the manufacturer's instructions. Results are expressed as the mean ± S.D. of 12 mice. *P < 0.05, compared with untreated control of tumor-transplanted animals (model group). The normal group indicates healthy animals without tumor xenograft for comparison.

The activation of NK cells is a good indicator of antitumor effect. After tumor-bearing mice were treated with liqi (50 g/kg), splenic lymphocytes showed more cytotoxicity than did splenocytes from untreated mice (*P *< 0.05) (Figure 4). These results indicate that liqi increased NK cell cytotoxicity.

### Effect of liqi on TNF-α activity in tumor-bearing mice

**Figure 5:**
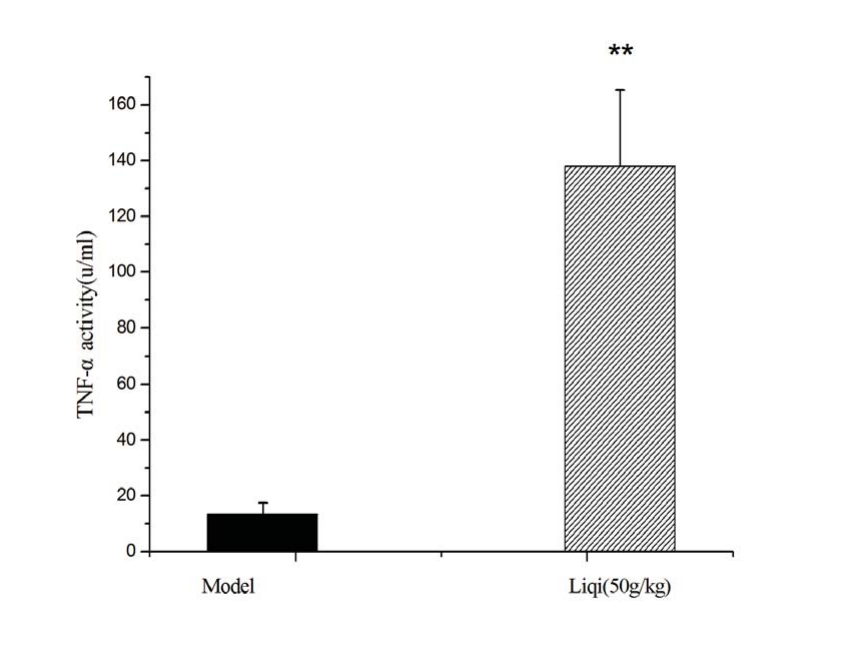
**Effect of Liqi on TNF-α activity in tumor-bearing mice**. Sarcoma 180 tumor cells were subcutaneously implanted (2 × 10^6 ^cells suspended in 50 μl of PBS) in the right axillary region of syngeneic BALB/c mice. After 24 h, Liqi (50 g/kg) was administrated daily by gavage for 12 days. Then tumor-bearing BALB/c mice received 20 μg/0.2 ml of LPS intravenously. After 1.5 h, blood samples were collected from the mice's orbital plexuses. TNF activity was determined by a cytotoxicity assay on L929 cells. The percent cytotoxicities were plotted against the logarithm of sample quantity. One unit of TNF activity was defined as the sample quantity required to achieve 50% cytotoxicity in the reaction. Results are expressed as the mean ± S.D. of 12 mice. ** P < 0.01, compared with untreated control of tumor-transplanted animals (model group).

We checked whether Liqi interfered in the LPS-induced TNF-α activity in tumor bearing mice. As shown in Figure 5, Liqi (50 g/kg) significantly increased TNF-α cytotoxicity on L929 cells (*P *< 0.01).

### Effect of liqi on metastasis of tumor cells to the lung

**Figure 6:**
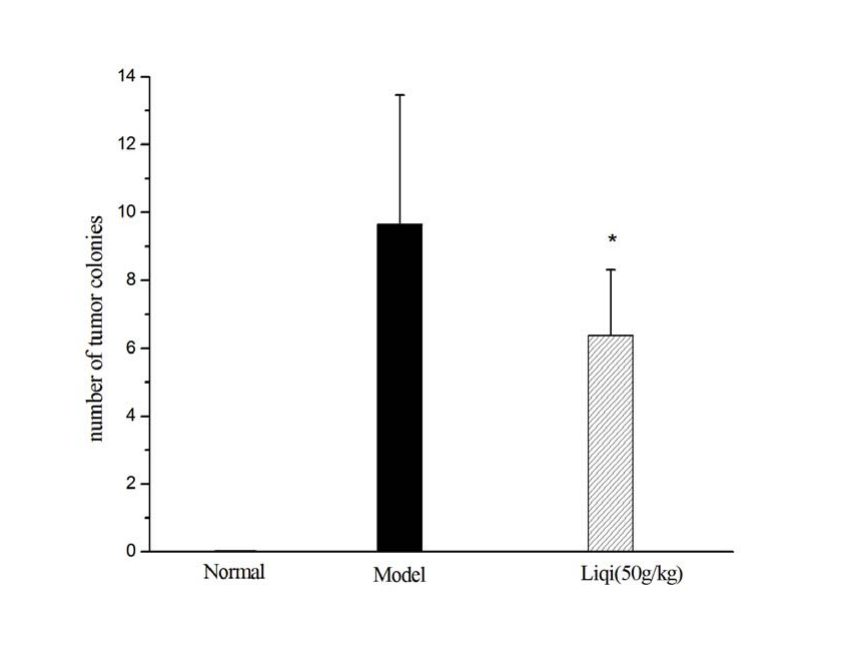
**Effect of Liqi on metastasis of tumor cells to the lungs**. Lewis lung carcinoma cells were subcutaneously implanted (2 × 10^6 ^LLC tumor cells suspended in 50 μl of PBS) in the right axillary region of syngeneic C57BL/6 mice. After 24 h, Liqi (50 g/kg) was administrated daily by gavage for 21 days. Lungs were removed and fixed in 4% formalin. Tumor colonies were counted under microscope (×200). Results are expressed as the mean ± S.D. of 12 mice. * P < 0.05, compared with model group. The normal group indicates healthy animals without tumor xenograft for comparison.

As shown in Figure 6, liqi prevented metastases to the lung. Fewer tumor colonies were observed in the lungs of mice treated with liqi compared with untreated mice (*P *< 0.05). The inhibition effect was 33.89%.

### Effect of liqi on platelet aggregation in tumor-bearing mice

**Figure 7:**
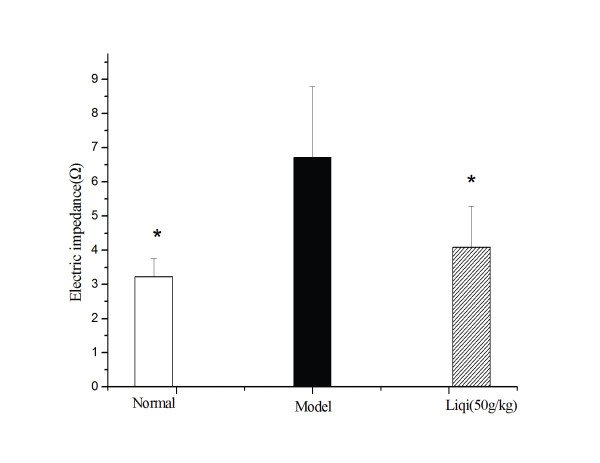
**Effect of Liqi on platelet aggregation in tumor-bearing mice**. Lewis lung carcinoma cells were subcutaneously implanted (2 × 10^6 ^LLC tumor cells suspended in 50 μl of PBS) in the right axillary region of syngeneic C57BL/6 mice. After 24 h, Liqi (50 g/kg) was administrated daily by gavage for 21 days. Platelet aggregation was monitored by measuring electric impedance using a whole-blood aggregometer. Results are expressed as the mean ± S.D. of 12 mice. *P < 0.05, compared with untreated control of tumor-transplanted animals (model group). The normal group indicates healthy animals without tumor xenograft for comparison.

As shown in Figure 7, increased platelet aggregation was observed in tumor-bearing mice compared with the normal group (*P *< 0.05); however, liqi (50 g/kg) significantly decreased platelet aggregation compared with untreated mice (*P *< 0.05).

### Effect of liqi on TXA_2 _and PGI_2 _in tumor-bearing mice

As shown in Table 4, TXB_2 _concentration was increased and 6-Keto-PGF_1α _was decreased in the blood plasma of tumor-bearing mice compared with normal group (*P *< 0.01); consequently, the ratio of TXB_2 _to 6-Keto-PGF_1α _was significantly increased compared with that of the normal group (*P *< 0.01). However, when the tumor-bearing mice were treated with liqi (50 g/kg), the level of TXB_2 _was markedly lowered, 6-Keto-PGF_1α _was raised, and the ratio of TXB_2 _to 6-Keto-PGF_1α_was significantly decreased compared with untreated mice (*P *< 0.01).

**Table 4:** Effect of Liqi on TXA_2 _and PGI_2 _in tumor-bearing mice.

**Groups**	**Doses (g/kg)**	**TXB_2 _(pg/ml)**	**6-Keto-PGF_1α _(pg/ml)**	**T/P**
Normal	0	1011.64 ± 102.57**	404.12 ± 51.33**	2.72 ± 0.78**
Model	0	1583.5 ± 102.45	178 ± 31.12	8.76 ± 1.67
Liqi	50	1185.43 ± 148.45**	323.15 ± 104.25**	4.81 ± 1.22**

Each group consists of 12 mice. Results are presented as mean ± S.D. **P < 0.01, compared with model group.

## Discussion

Our findings suggest that liqi has a significant anti-tumor effect against Sarcoma 180 tumor and Lewis lung carcinoma, which is likely because of its immune-modulating effects. Liqi enhanced NK cell activity, increased the Th/Ts ratio, stimulated the production and activity of IL-2 in splenocytes, and increased TNF-α activity in serum. Moreover, liqi inhibited the Lewis lung carcinoma metastasis, which might be related to its inhibition of platelet aggregation and normalization of the balance between TXA_2 _and PGI_2_. Furthermore, liqi inhibited the human gastric carcinoma growth in xenografted mice. We demonstrated that liqi has an anti-tumor effect and this effect may be related to immune regulation and anticoagulation.

Previous reports have shown that the constituents of liqi had an anti-tumor effect in vitro. We have shown that treatment with liqi inhibited tumor growth in vivo. We found that liqi arrested the proliferation of the human gastric carcinoma cell line SGC-7901 from xenografted mice in the G0/G1 phase and reduced the proportion of cells in the S and G2/M phases (mitotic/dividing phases).

Although the mechanisms underlying liqi's anti-tumor effect may be complex, our findings demonstrated that this effect was most likely related to immune regulation. Host immunity is implicated in the development and progression of malignant disease. Mice with tumors show a change in T lymphocyte subpopulations and NK cell activity. T cells can affect tumor cells directly or can act indirectly by producing cytokines that amplify cytotoxic T lymphocyte responses or activate NK cells and macrophages. Experimental studies have outlined the critical role of CD4+ and CD8+ T cells and NK cells in mediating anti-tumor immunity [17-20]. The present study confirms the decrease in the ratio of CD4+/CD8+ with increasing tumor burden, which appeared to result mainly from a decrease in CD4+ T cells and an increase in CD8+ T cells. However, the results also showed that the number of CD4+ T cells and the CD4+/CD8+ ratio increased significantly after administration of liqi, while the number of CD8+ cells decreased. This finding indicates that liqi can regulate the disordering of T lymphocyte subsets and improve the immune response.

We also found that administering liqi increased the activity of NK cells in tumor-bearing mice. NK cells are the major mediators of the innate anti-tumor immune responses; they eradicate tumors by recognizing stress-inducible ligands on tumors and execute tumor cells with perforin and granzyme in vivo [21]. NK cells eradicate solid tumors by apoptosis [21]. Liqi augments the cytotoxic potential of NK cells in vivo. Our data show that liqi reversed tumor-mediated suppression of NK cell cytotoxicity.

We found that the anti-tumor activity of liqi might also be related to its regulatory effects on IL-2 and TNF-α, both of which play important roles in cancer therapy [22,23]. IL-2 has an anti-tumor effect because of its immune-regulating function [24], TNF-α had an anti-tumor activity against sarcoma 180. In addition to direct cytotoxicity against tumor cells, TNF induced a host-mediated factor which contributed to the anti-tumor effects [25]. Rychly J et al. [26] also demonstrated that TNF-α induced strong necrosis in sarcoma 180 in vivo and showed total regression. The infiltration of inflammatory cells was observed in the sarcoma 180 tumor. Their result suggested that cell infiltration may be of importance for tumor regression. A synergistic anti-tumor effect has been observed when TNF-α is combined with IL-2 [27]. We demonstrate that liqi can stimulate the production and activity of IL-2 and TNF-α in mouse Sarcoma 180 tumor-bearing mice.

Furthermore, we demonstrated that liqi inhibited metastasis of Lewis lung carcinomas. Metastasis is the major cause of death from cancer. Tumor-induced platelet aggregation is believed to protect tumor cells from immunological assault in circulation [28]; platelets protect tumors from TNF-α-mediated cytotoxicity [29]. Platelets also facilitate the adhesion of tumor cells to the vascular endothelium and release a number of growth factors that promote tumor growth [30]. Recently, it has been reported that platelets contribute to tumor-induced angiogenesis by releasing angiogenic growth factors, such as vascular endothelial growth factor [31]. Our results demonstrated that platelet aggregation was increased in C_57_BL mice with Lewis lung carcinomas, and liqi inhibited platelet aggregation in these mice.

PGI_2_, a potent vasodilator and inhibitor of platelet aggregation, is an important antithrombotic mediator in vivo [32]. It also inhibits carcinogenesis [33]. Conversely, TXA_2 _stimulates platelet aggregation and amplifies the response to other platelet agonists [34]. We demonstrated that the level of PGI_2 _in serum was decreased and the level of TXA_2 _was increased, which resulted in an increased T/P ratio in C_57_BL mice with Lewis lung carcinomas. However, Liqi treatment inhibited the increase in the T/P ratio.

## Conclusion

In summary, we demonstrated for the first time that liqi has a significant inhibitory effect on tumor growth in vivo. This inhibition may be related to the immunoregulatory effects of liqi, such as stimulating IL-2 and TNF-α production and activity and increasing the ratio of CD4+ to CD8+ T lymphocytes and the activity of NK cells. These data lead us to propose that the anticancer function of liqi is underpinned by the improvement of immune function. Furthermore, liqi has a significant inhibitory effect on tumor metastasis, which is most likely related to its anticoagulation effects. These findings provide an experimental basis for the use of liqi for tumor therapy. The findings of our study may shed light on the pharmacologic basis for the clinical application of traditional Chinese medicine in treating cancer.

## Competing interests

The authors declare that they have no competing interests.

## Authors' contributions

DBJ carried out the studies, performed the statistical analysis and drafted the manuscript. JY carried out the study design, participated in the studies and drafted the manuscript. BWQ conceived of the study and participated in its design and coordination. YMJ participated in cell cycle and peripheral blood T lymphocyte subsets analysis. All authors read and approved the final manuscript.

## Pre-publication history

The pre-publication history for this paper can be accessed here:


